# Clinical and demographic moderators of self‐care and hospitalizations in pre‐coronary artery bypass grafting patients: A cross‐sectional study

**DOI:** 10.1111/jocn.17381

**Published:** 2024-08-06

**Authors:** Naruebeth Koson, Nittaya Srisuk, Nipaporn Juntratip, Piyatida Borvornsudhasin, David R. Thompson, Chantal F. Ski

**Affiliations:** ^1^ Boromrajonnani College of Nursing Nakhon Si Thammarat, Faculty of Nursing Praboromarajchanok Institute Nakhon Si Thammarat Thailand; ^2^ Faculty of Nursing Rajamangala University of Technology Thanyaburi Khlong Hok Thailand; ^3^ Cardiovascular and Thoracic Intensive Care Unit, Suratthani Hospital Surat Thani Thailand; ^4^ School of Nursing and Midwifery Queen's University Belfast Belfast UK; ^5^ Australian Centre for Heart Health Deakin University Melbourne Victoria Australia

**Keywords:** coronary artery bypass grafting, hospitalizations, self‐care

## Abstract

**Aim:**

To examine the influence of clinical and demographic factors on self‐care behaviour and hospitalization rates among patients with coronary heart disease awaiting coronary artery bypass grafting.

**Background:**

Appropriate self‐care behaviour can improve the management of patients with coronary heart disease and reduce hospitalization rates among those awaiting coronary artery bypass graft surgery. However, little is known about the influence of clinical and demographic factors on self‐care or hospitalizations in this population.

**Design:**

A cross‐sectional study.

**Methods:**

A convenience sample of 99 participants diagnosed with coronary heart disease awaiting coronary artery bypass grafting surgery were recruited from an outpatient clinic of a public tertiary hospital in southern Thailand. Data were collected on clinical (left ventricular ejection fraction, symptom severity and comorbid disease) and demographic (age, education level and marital status) factors, self‐care behaviour and hospitalization rates. Path analysis using LISREL was performed to examine the influence of self‐care on hospitalizations, with clinical and demographic factors as moderators.

**Results:**

Path analysis showed that clinical and demographic factors accounted for nearly half of the variance (46%) in self‐care, and that self‐care accounted for nearly half of the variance (48%) in hospitalization rates.

**Conclusion:**

Our findings demonstrate that clinical and demographic factors play an important role in self‐care behaviour, and in turn hospitalization rates of pre‐coronary artery bypass graft surgery patients. It is suggested that the period pre‐surgery is an ideal time to introduce programmes designed to bolster self‐care and minimize uncertainty among this patient population and that nurses are well‐positioned to do so.

**Reporting Method:**

Study methods and results reported in adherence to the STROBE checklist.

**Patient or Public Contribution:**

Patients contributed their consent, time and data to the study.

**What does this paper contribute to the wider global community?**

- This paper provides evidence that clinical and demographic factors impact self‐care behaviour and act as moderators of hospitalization rates among individuals with coronary heart disease awaiting cardiac surgery.
- It also highlights that the pre‐surgery waiting list period provides an ideal opportunity for nurses to provide patient education and skills training designed to bolster self‐care behaviour, minimize uncertainty and reduce the risk of hospitalization.

## 1 INTRODUCTION

Coronary heart disease (CHD) affected over 244 million people worldwide in 2020, with the highest prevalence rates observed in North Africa, the Middle East, Eastern Europe and Central and South Asia (Tsao et al., 2022). Over the past two decades, age‐standardized death rates across South, East and South East Asia have been increasing (Roth et al., 2020). In Thailand, CHD is the primary cause of premature mortality, a trend that is expected to continue (Ministry of Public Health Thailand, 2021). An effective treatment for extending and enhancing the life of patients with CHD is coronary artery bypass grafting (CABG) surgery (Doenst et al., 2019). A recent report by the Society of Thoracic Surgeons of Thailand shows increasing numbers of patients undergoing CABG in government and private hospitals (Society of Thoracic Surgeons of Thailand, 2022). Of note, patients with CHD often experience significant wait times for CABG surgery due to resource limitations, such as a shortage of ICU beds, operating rooms and cardiothoracic surgeries, a situation that has been exacerbated due to COVID‐19 (Cesena et al., 2004).

Patients experiencing prolonged waiting times for CABG are at increased risk of death and morbidity (Rexius et al., 2004, 2005). Prolonged waiting periods for surgery have the potential to compromise cardiovascular function due to heightened myocardial ischemia caused by narrowed coronary arteries. For example, patients with CHD awaiting CABG have been shown to have an average waiting time of 55 days, and this is associated with higher mortality rates (Koomen et al., 2001). In Thailand, the waiting time for CABG ranges from 69 to 757 days (Indratula et al., 2013). In this respect, the pre‐CABG waiting period poses a critical and vulnerable phase coinciding with disease progression, and potential for cardiac dysfunction or injury. During this time, there is potential for patients to encounter significant major adverse cardiovascular events (MACE), including unstable angina, dyspnea, fatigue, weakness and shortness of breath (Banner, 2010; Bengtson et al., 1996; Morgan et al., 1998; Sampalis et al., 2001). Further, in rural Thailand there is limited availability of cardiac rehabilitation programs. These findings underscore the uncertainty of the waiting period, which also gives rise to psychological and social challenges. Importantly, this period also provides an opportunity to intervene and limit or prevent the occurrence of MACE (Golaghaie et al., 2019; McCann et al., 2019).

Nurses play an important role in the prevention and management of CHD, including educating and supporting patients in self‐care behaviours such as unhealthy lifestyle modification, medication adherence, and symptom monitoring (Jia et al., 2020; Knuuti et al., 2020; Pizga et al., 2022; Riegel et al., 2017). Effective self‐care has been shown to reduce cardiovascular risk and the use of medical services, and increase quality of life (Knuuti et al., 2020; Lee et al., 2022; Li et al., 2021; Riegel et al., 2017). Currently in Thailand self‐care instruction is delivered to patients verbally primarily by nurses and also surgeons on the first day of registration on the CABG waiting list at the cardiothoracic outpatient clinic. This self‐care information is received together with several other pieces of important information including, worsening cardiac symptoms, preparation for admission for CABG surgery, in‐hospital post‐CABG care. As such, the waiting period for CABG is an ideal time to introduce interventions specifically designed for optimal self‐care to reduce risk of hospitalization and adverse effects both before and after CABG (Furze et al., 2009; Goodman et al., 2008; Kotb et al., 2014; Waite et al., 2017).

Self‐care is described as the process of maintaining health through health‐promoting practices and managing illness (Riegel et al., 2012). It consists of three components: self‐care maintenance, self‐care monitoring and self‐care management. Self‐care maintenance involves patient behaviour aimed at preserving physical and emotional stability. Self‐care monitoring entails being vigilant for changes in signs and symptoms, whilst self‐care management involves responding to symptoms when they occur (Riegel et al., 2012). Examining the theoretical connections between factors with potential to influence self‐care can help to better understand the multi‐dimensionality of self‐care (Riegel et al., 2019). Further, clinical factors such as LVEF, symptom severity and comorbid diseases and demographic ones such as gender, age, marital status and educational level play a significant role in determining self‐care behaviour and subsequent health outcomes (Koomen et al., 2001; Morgan et al., 1998; Rexius et al., 2004). Importantly, cultural differences and social factors must be taken into account when evaluating self‐care (Osokpo & Riegel, 2021; Polsook & Aungsuroch, 2021; Riegel et al., 2019). Although a few studies have been conducted in Thailand that have explored various factors related to patient symptoms and experiences among individuals awaiting CABG (Indratula et al., 2013; Utriyaprasit, 2016), none have examined the influence of clinical and demographic factors on self‐care behaviour and their potential contribution to hospitalizations.

Thus, in patients with CHD awaiting CABG in Thailand, the aim of this study was to investigate the influence of self‐care behaviour on hospitalizations, with demographic and clinical factors as moderators. The objective of this study was twofold: (1) to describe self‐care behaviour and (2) to examine the factors that influence self‐care behaviour and hospitalizations.

## 2 METHODS

### 2.1 Study design

A descriptive cross‐sectional design was used. Patients were recruited from the outpatient clinic of an 800 bed public tertiary hospital with a cardiac centre in southern Thailand specializing in cardiovascular revascularization procedures such as percutaneous coronary intervention and CABG. Data were collected between April and September 2021.

### 2.2 Sample

A convenience sample of patients with CHD was enrolled in the study. Inclusion criteria were: (a) age 18 years or older; (b) documented clinical diagnosis of CHD, listed as candidates for CABG surgery on the waiting list, with a waiting time for surgery exceeding 3 months; (c) undergoing CABG for the first time; and (d) able to provide informed consent. Exclusion criteria were: patients with cognitive dysfunction, mental illness or frailty (as per documented medical history), and those with communication difficulties. The sample size for the study was calculated to be 99 patients with CHD waiting to undergo CABG surgery. This determination considered six clinical and demographic moderators of self‐care, with a 95% degree of confidence, a 5% margin of error, an effect size of .15, and a test power of .80 (Cohen, 2013).

### 2.3 Materials

#### 2.3.1 Demographic characteristics

Patient characteristics were collected using a self‐report sociodemographic questionnaire (e.g. age, education level [primary school or less, secondary school, or college/higher], and marital status [married, or single and separated]).

#### 2.3.2 Clinical characteristics

Clinical characteristics were collected from the hospital standard medical records and included data on LVEF, severity of symptoms and comorbid diseases. LVEF was recorded by a cardiologist; severity of symptoms was assessed using the Canadian Cardiovascular Society Classification for Angina (CCSC): Class I—angina experienced only during strenuous or prolonged physical activity; Class II—angina experienced during vigorous physical activity with slight limitation; Class III—symptoms occur with daily living activities, resulting in moderate limitation; and Class IV—inability to perform any activities, with or without angina, even at rest (Sangareddi et al., 2004). A higher CCSC class is significantly associated with an increased incidence of all‐cause hospitalizations (Sangareddi et al., 2004); and comorbidity was evaluated using the Charlson Comorbidity Index (CCI), which uses an interview format covering 19 medical conditions. In this study, myocardial infarction, the primary diagnosis, was removed from the list and hypertension was added as the main comorbidity associated with myocardial infarction. Scores on the CCI range from 0 (no comorbidities) to 1, 2, 3 or 6, depending on the severity of the illness category. The total score can range from 0 to 37 (Charlson et al., 1987). It has been reported that an increased CCI score is associated with a higher risk of 5‐year mortality and 1‐year readmission (Charlson et al., 1987).

#### 2.3.3 Self‐care behaviour

Self‐care behaviour was evaluated using the Self‐Care of Coronary Heart Disease Index version 3 (SC‐CHDI v.3) (Dickson et al., 2017), which consists of 23 items divided into three scales: Self‐Care Maintenance (SCMT) scale, which assesses the frequency of behaviour such as keeping healthcare appointments, taking medications, and engaging in physical activity; Self‐Care Monitoring (SCMN) scale, which evaluates the frequency of behaviour such as monitoring the condition, assessing feelings, and measuring blood pressure; and Self‐Care Management (SCMM) scale, which assesses the likelihood of engaging in behaviour such as adjusting activity levels, taking medication, and reporting symptoms to healthcare providers when experiencing symptoms. Each item on the three scales is rated on a 1‐to‐5 Likert scale. The SC‐CHDI v3 scoring guidelines use a standardized score that is calculated independently for each scales. First, a raw scale score is calculated, and then it is transformed into a standardized score ranging from 0 to 10. To assess whether patients had appropriate self‐care, a cutoff score of ≥70, indicating ‘adequate’ self‐care behaviour (Cilli et al., 2022; Dickson et al., 2023). The Thai version of SC‐CHDI v3 has been translated and published elsewhere with demonstrated acceptable internal consistency; Cronbach's alpha and McDonald's omega coefficients ranging from .821 to .910 (Koson et al., 2023).

#### 2.3.4 Hospitalizations

For the purpose of this study, hospitalization was defined as the occurrence of patients with CHD on the waiting list for CABG surgery being admitted to the hospital within 3 months of their initial diagnosis. The data were obtained from the patients' medical records; if unrecorded, patients were contacted by telephone.

### 2.4 Data collection

Patients who met the inclusion criteria were provided written informed consent after being informed about the study's purpose, conduct, expected benefits and potential risks. Patients were also informed of their right to withdraw at any time during the study. Once permission was granted, patients were asked to complete a set of paper‐based self‐administered questionnaires. For patients who could not read unaided, a nurse read out the questions and entered the responses on their behalf. The questionnaires were provided either before or after their visit to the physician in the outpatient clinic and took approximately 30 min to complete. Following that, hospitalizations were monitored monthly for 3 months during the study period. In cases where the health status of a patient was not known from their medical history, they were contacted by telephone.

### 2.5 Statistical analyses

The Statistical Package for the Social Sciences (SPSS) program version 23 was used for data management and analysis. Single‐item variables, such as age, educational level, marital status, LVEF, CCSC and CCI, were coded as raw data. All assumptions for multivariate analyses, including normality, homoscedasticity, linearity, and multicollinearity, were assessed and met. Zero‐order correlations among predictor variables ranged from −.01 to .65, indicating the absence of multicollinearity. Bivariate Pearson's coefficient was then used to analyse the correlation between variables: a strong correlation is indicated if the coefficient value lies between ±.50 and ±1; a medium correlation is indicated if the value lies between ±.30 and ±.49; and a small correlation is indicated when the value lies below ±.29 (Cohen, 2013).

Path analysis was conducted using linear structural relationship (LISREL), with significance accepted at the *p* < .05 level. The following fit indices and criteria were evaluated using commonly accepted thresholds for acceptable fit (Hair Jr. et al., 2009; Schermelleh‐Engel et al., 2003): Goodness of Fit Index (GFI; >.90), Adjusted Goodness of Fit Index (AGFI; >.85), Root Mean Square Error of Approximation (RMSEA; <.08), and Standardized Root Mean Square Residuals (SRMR; <.10). CMIN/df was used to assess the fit between the theoretical model and sample data, with a good fit indicated when CMIN/df is <2 (Hair Jr. et al., 2009; Schermelleh‐Engel et al., 2003). Once the hypothesized model fit the data, associations between variables were calculated, and the path coefficients and *R* ^2^ were evaluated. Goodness‐of‐fit indices were used to determine whether the model fit the data effectively.

### 2.6 Ethical considerations

The guidelines of the Declaration of Helsinki were followed. Ethical approval for this study was granted by the Institutional Review Board of Suratthani Hospital (95/2563). Participants received a thorough explanation of the research objectives, methods, anticipated benefits, possible risks, and their opportunity to withdraw from the study at any time before any data were collected. For those who agreed to participate, written informed consent was obtained. They were also informed that all collected data would be safely stored; all data were encrypted and coded before being securely stored in a locked cabinet or with restricted access available only to the study team. According to IRB regulations, after the article is published, data will be stored for 1 year for further inspection. Subsequently, the questionnaire will be discarded.

## 3 RESULTS

### 3.1 Participant characteristics

A total of 99 patients consented to participate in the study. Almost three‐quarters of participants were men (71.7%), with an age range of 55–76 years old and a mean age of 63 years. Nearly half of the participants had a primary school education or less (42.4%), and most were married (67.7%). Participants LVEF ranged from 36 to 74 percent, with a mean of approximately 60 percent (SD = 12.43). Symptom severity as per the CCSV was: 44.4% Class I; 36.4% Class II, and 19.2% Class III or IV. Comorbidity as per the CCI ranged from 1 to 6, with a mean value of 2.06 (SD = 1.35), indicating relatively low comorbid disease. Additionally, one quarter of participants were hospitalized within 3 months (24.2%), as shown in Table 1.

**TABLE 1:** Demographic and clinical characteristics of the sample (*n* = 99).

Characteristic	Number	Percentage
Gender		
Male	71	71.7
Female	28	28.3
Age, year, mean ± SD (63.37 ± 6.66)		
<60	33	33.3
≥60	66	66.7
Education level		
Primary school or less	42	42.4
High school	31	31.3
Higher education	26	26.3
Marital status		
Single	14	14.1
Married	67	67.7
Divorced	18	18.2
LVEF, percentage, mean ± SD (6.06 ± 12.43)		
LVEF <40	19	19.2
LVEF ≥40	80	8.8
Severity of symptoms		
CCSC class I	44	44.4
CCSC class II	36	36.4
CCSC class III–IV	19	19.2
Comorbidities		
Low	71	71.7
Moderate	28	28.3
High	0	0
Hospitalization		
No	75	75.8
Yes	24	24.2

Abbreviations: CCSC, Canadian Cardiovascular Society Classification for Angina; LVEF, left ventricular ejection fraction; SD, standard deviation.

### 3.2 Self‐care behaviour

Participants demonstrated “adequate self‐care behavior” scores (mean ≥ 70) for self‐care maintenance 72.33 (SD = 1.51) and for self‐care monitoring 71.10 (SD = 13.12), whilst self‐care management showed “inadequate self‐care behavior” 67.56 (SD = 16.92). Descriptive statistics for the three self‐care subscales are summarized in Table 2.

**TABLE 2:** Descriptive statistics for individual items of self‐care maintenance, self‐care monitoring and self‐care management scales.

	Mean	SD	Skewness	Kurtosis	Self‐care behaviour
Self‐care maintenance	72.33	1.51	.04	−.92	Adequate
Take aspirin or other blood thinner	9.10	13.51	−1.23	1.12	Adequate
Take prescribed medicines without missing a dose	89.09	14.92	−.97	−.52	Adequate
Eat fruits and vegetables	86.26	17.06	−1.16	.89	Adequate
Keep appointments with your healthcare provider	82.83	21.95	−.99	−.06	Adequate
Avoid cigarettes and/or smokers	77.37	24.01	−.72	−.56	Adequate
Try to avoid getting sick (e.g. flu shot, wash your hands)	75.96	17.61	−.41	−.43	Adequate
Ask for low fat items when eating out or visiting others	71.11	18.56	−.09	−.81	Adequate
Do something to relieve stress (e.g. medication, yoga, music)	71.11	15.96	−.01	.24	Adequate
Do physical activity (e.g. take a brisk walk, use the stairs	56.97	22.56	.91	.24	Inadequate
Self‐care monitoring	71.10	13.12	−.81	.11	Adequate
Check your blood pressure	79.80	17.73	−.52	−.50	Adequate
Monitor for symptoms	78.59	18.79	−.46	−.73	Adequate
Pay attention to changes in how you feel	77.98	15.78	−.20	−.56	Adequate
Monitor for medication side‐effects	77.98	15.52	−.62	.41	Adequate
Monitor your weight	75.56	19.70	−.32	−.91	Adequate
Monitor your condition	75.35	16.37	−.23	−.43	Adequate
Monitor whether you tire more than usual doing normal activities	72.93	16.49	−.59	−.13	Adequate
Self‐care management	67.56	16.92	−.79	−.21	Inadequate
Tell your healthcare provider about the symptom at the next office visit	81.41	19.64	−.80	−.38	Adequate
Take a medicine to make the symptom decrease or go away	77.98	2.30	−.51	−.86	Adequate
Take an aspirin	7.91	17.67	−.50	−.58	Adequate
Call your healthcare provider for guidance	69.90	24.31	−.37	−.86	Inadequate
Change your activity level (slow down, rest)	68.89	2.25	.09	−1.07	Inadequate
Did the treatment you used make you feel better	68.69	24.27	−.37	−.94	Inadequate

Abbreviation: SD, standard deviation.

For self‐care maintenance, taking aspirin or other blood thinner (9.10 ± 13.51) and taking prescribed medicines without missing a dose (89.09 ± 14.92) were scored highest, whilst to do something to relieve stress (e.g. medication, yoga, music) and ask for low‐fat items when eating out or visiting others were scored lowest. Physical activity (e.g. taking a brisk walk, using the stairs) was the only self‐care maintenance item to indicate inadequate self‐care behaviour (mean < 70).

For self‐care monitoring all items indicated adequate self‐care behaviour scores. The two highest scored self‐care monitoring items were check your blood pressure and monitor for symptoms, 79.80 ± 17.73, and 78.59 ± 18.79 respectively. Monitoring whether you tire more than usual doing normal activities was the lowest scoring item.

For self‐care management highest to lowest adequate scores was, telling your healthcare provider about the symptom at the next office visit (81.41 ± 19.64), taking medicine to make the symptom decrease or go away (77.98 ± 2.30), and taking an aspirin (7.91 ± 17.67). Items scoring inadequate self‐care management were, change your activity level (slow down, rest), call your healthcare provider for guidance, and did the treatment you used to make you feel better.

### 3.3 Correlations of study variables

Bivariate Pearson's correlations were used to assess associations between demographic factors (age, education level and marital status), clinical factors (LVEF, severity of symptom, comorbid disease), self‐care behaviour and hospitalizations. For demographic factors, education level had a medium positive correlation with self‐care maintenance (*r* = .33, *p* < .001) and had small positive correlation with self‐care monitoring (*r* = .28, *p* < .001), marital status had a small to medium positive correlation with all three self‐care behaviours. For clinical factors, LVEF had a small to medium positive correlation with all self‐care behaviours, and a medium negative correlation with hospitalization (*r* = −.34, *p* < .001), severity of symptoms had a small negative correlation with self‐care monitoring (*r* = −.29, *p* < .001), and comorbid disease had a medium negative correlation with self‐care maintenance (*r* = − .31, *p* < .001), and small negative correlation with self‐care management (*r* = − .22, *p* < .05). All self‐care behaviours (maintenance, monitoring and management) were significantly moderate positive intercorrelated. Further, self‐care maintenance (*r* = − .51, *p* < .05), self‐care monitoring (*r* = − .65, *p* < .05), self‐care management (*r* = − .62, *p* < .05) strong negative correlation with hospitalization (Table 3).

**TABLE 3:** Correlation among demographic factors, clinical factors, self‐care behaviours, and hospitalizations.

	Age	Edu	MS	LVEF	SoS	Comd	SCMT	SCMN	SCMM	Hosp
Demographic factors										
Age	1									
Edu	−.21*	1								
MS	.10	.45**	1							
Clinical factors										
LVEF	.05	.02	.09	1						
SoS	−.27**	−.10	−.06	−.22*	1					
Comd	.17	−.24*	−.15	.02	−.01	1				
Self‐care behaviours										
SCMT	−.14	.33**	.31**	.31**	−.09	−.31**	1			
SCMN	.04	.28**	.26*	.26**	−.29**	−.18	.40**	1		
SCMM	.11	.10	.20*	.36**	−.12	−.22*	.49**	.51**	1	
**Hosp**	.14	−.14	−.11	−.34**	.13	.01	−.51**	−.65**	−.62**	1

Abbreviations: Comd, Comorbid disease; Edu, Education level; Hosp, Hospitalizations; LVEF, Left ventricular ejection fraction; MS, Marital status; SCMM, Self‐care management, SCMN, Self‐care monitoring; SCMT, Self‐care maintenance; SoS, Severity of symptoms.

* *p* < .05.

** *p* < .01.

### 3.4 Factors influencing self‐care and hospitalizations

Results from the hypothetical model matched the empirical evidence. Self‐care behaviour comprising three dimensions of maintenance, monitoring and management was explained by: demographic factors (age, education level, marital status) and clinical factors (LVEF, severity of symptom and comorbid diseases) that accounted for 46% (*R* ^2^ = .46) of the variance as shown in Table 4. The model of demographic and clinical factors as moderators of self‐care behaviour yielded consistent results with the empirical data and contributed to explaining 48% (*R* ^2^ = .48) of the variance in hospitalizations as shown in Table 4.

**TABLE 4:** Proportion of variance accounted for by the model.

Key factors	Influencing factors	*R* ^2^
Self‐care behaviours^a^	Age	.46
	Education level	
	Marital status	
	Left ventricular ejection fraction	
	Severity of symptoms	
	Comorbid disease	
Hospitalization^b^	Self‐care behaviours	.48

^a^ Self‐care behaviour was explained by age, educational level, marital status, and clinical factors including left ventricular ejection fraction, severity of symptoms, and comorbid diseases which accounted for 46% of the variance.

^b^ The model incorporating demographic and clinical factors as moderators of self‐care behaviour and self‐care behaviour accounted for 48% of the variance in hospitalization rates.

### 3.5 Path analysis model

**FIGURE 1:**
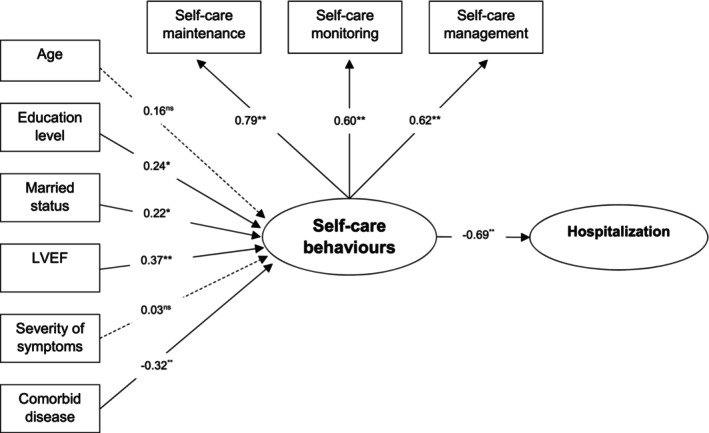
Final model of factors predicting self‐care behaviour and hospitalizations among patients waiting for CABG. Model fit indices: (*χ* ^2^= 1.68; df = 11; *p* = .47/*χ* ^2^df = .97; GFI = .98; AGFI = .90; RMSEA = .00; SRMR = .04).

The path analysis model demonstrated a good fit index (*χ* ^2^ = 1.68, df = 11, *p* = .47, *χ* ^2^/df = .97, GFI = .98, AGFI = .90, RMSEA = .00, and SRMR = .04), as illustrated in Figure 1. The final model revealed that education level (*β* = .24, *p* < .05), marital status (*β* = .22, *p* < .05), and LVEF (*β* = .37, *p* < .001) had a significant direct positive effect on self‐care behaviour. Conversely, comorbid disease had a direct negative effect on self‐care behaviour (*β* = −.32, *p* < .001). Age and severity of symptoms did not have a significant effect on self‐care behaviour. Lastly, self‐care behaviour demonstrated a direct negative effect on hospitalization (*β* = −.69, *p* < .001).

## 4 DISCUSSION

This study demonstrates the influence of clinical and demographic factors on self‐care behaviour of patients with CHD awaiting CABG in Thailand, and the subsequent contribution of self‐care to hospitalizations. These findings align with the model of the middle range theory of self‐care of chronic illness (Riegel et al., 2012, 2019) through confirming the important role of clinical factors in relation to self‐care behaviour, and providing insights into the influence of demographic factors on self‐care in a pre‐CABG population.

Overall, our findings indicate that self‐care behaviours of maintenance and monitoring were adequate among patients awaiting CABG surgery, though self‐care management was inadequate. When considering the specific self‐care behaviour of patients with inadequate self‐care practices, there were significant similarities: patients demonstrated inadequate self‐care maintenance in terms of engaging in physical activity (e.g. taking brisk walks or using stairs), inadequate self‐care monitoring to assess whether they experienced more fatigue than usual during routine activities, and inadequate self‐care management, such as adjusting their activity levels (e.g. slowing down or resting), seeking guidance from healthcare providers, and evaluating the effectiveness of their treatment. Rattanaprom et al. (2023) found inadequate self‐care practices in patients with CHD who had undergone PCI in Thailand. Our findings concur, in part, that is physical activities. It was observed that these insufficient self‐care behaviours related to physical activity closely resembled the findings of a study on CHD patients in Jordan (Al‐Sutari & Ahmad, 2022), which appears to be a global issue among CHD patients (Gabulova et al., 2023; Kotseva et al., 2019).

Clinical and demographic factors, such as LVEF and comorbid disease, and educational level and living with a spouse, are correlated with self‐care behaviour (Babygeetha & Devineni, 2024; Rattanaprom et al., 2023; Riegel et al., 2017). Our study also confirmed that self‐care behaviour in all three dimensions are closely related, as proposed in the theory of self‐care (Riegel et al., 2012, 2019). Patients who practice self‐care have a lower likelihood of hospitalization compared to those with poor self‐care practices. In this study, 24% of the sample group were hospitalized within 3 months, similar to a study in Brazil that found 21% of patients with CHD experienced hospitalization (Oliveira et al., 2019). These findings are similar to studies in Australia and New Zealand (Labrosciano et al., 2017). The reasons for hospitalization are diverse. In Thailand, social support appears to be the most significant factor in predicting hospitalization in patients with CHD (Polsook & Aungsuroch, 2021). However, it is worth noting that more than half of the sample in this study was married. Exploring specific types of support that can help improve self‐care and reduce hospitalization is recommended (Babygeetha & Devineni, 2024). Pre‐operative education programs or perceived social support for patients awaiting CABG surgery could play a substantial role in social welfare and cardiac rehabilitation initiatives aimed at enhancing the quality of life for patients both before and after their CABG procedure (Jamaludin & Chan, 2019).

Self‐care was identified as a significant predictive factor for hospitalization. These findings shed some light on the factors that influence self‐care behaviour in patients during the waiting period for CABG surgery, primarily educational level, marital status, and LVEF, the severity of symptoms, and comorbidities. We also found inadequate self‐care maintenance and management in terms of engaging in physical activity as recommended in clinical guidelines. Barriers to exercise as part of self‐care for CHD patients persist. The limited availability of cardiac rehabilitation facilities in rural areas of Thailand (with concentration in Bangkok) poses significant obstacles to patients acquiring optimal self‐care skills. Further, patients receive all information relating to their CABG including self‐care instruction in a single day as an outpatient. Participants then return home and wait until they received notification for admission to prepare for CABG surgery.

Our findings are aligned with other recent studies including a longitudinal observational study, which found inadequate self‐care management and adequate self‐care maintenance for the self‐care trajectories of 430 patients with CAD (Ingadóttir et al., 2024). Despite slight improvements in self‐care maintenance over time, self‐care management either remained at a suboptimal level or decreased. Importantly, not attending rehabilitation (OR 2.175; CI 1.020–4.637, *p* = .044) predicted inclusion in the inadequate and worsening self‐care trajectory (Ingadóttir et al., 2024). Recommended was that interventions specifically supporting self‐care are likely to benefit patient outcomes. Further, Yuroong et al. (2021) conducted an interventional study in Thailand that assessed the effectiveness of a self‐care focused transitional care program among patients awaiting CABG found higher cardiac self‐efficacy in the intervention group. The authors concluded that during the waiting period for CABG nurses are in a pivotal position to make care transitions safer, with potential to enhance positive outcomes regarding patients awaiting elective cardiac surgery (Yuroong et al., 2021). Together, these findings demonstrate the importance of educating patients and promoting optimal self‐care behaviours whilst patients await CABG, and how nurses are well‐positioned to do so.

Our study is not without limitations. Whilst the sample group obtained from the statistical software tool was considered adequate when compared to other closely related research, the sample size appears relatively small. Caution should be taken when applying these results to similar patients in other geographical locations. Our findings may not be representative of the broader population, as the study is confined to a single centre and may not capture regional variations and length of time waiting for CABG was not accounted for in the analyses. We recommend examination of a broader range of variables in future research to enhance overall understanding, i.e. length of time waiting for CABG, symptoms interpretation, cultural beliefs, cognitive and functionalities. Furthermore, this study highlights the importance of adhering to guidelines that recommend exercise and cardiac rehabilitation for patients with CHD in Thailand. The limited availability of cardiac rehabilitation facilities in rural Thailand poses significant obstacles. However, home‐based preoperative exercise with caregiver support is likely beneficial for patients awaiting surgery.

## 5 CONCLUSION

Clinical and demographic factors play an important role in self‐care behaviour and act as moderators of hospitalization rates among pre‐CABG patients. Clinical factors such as comorbidities as classified by CCI were associated with inadequate self‐care behaviours and higher hospitalization rates and demographic factors such as lower education levels were associated with inadequate self‐care behaviours. Specifically, the self‐care management was sub‐optimal, particularly related to physical activities. Early identification of patients at risk of inadequate self‐care affords the opportunity for interventions to be delivered by nurses during a vulnerable time for pre‐CABG patients to improve self‐care behaviour and minimize the risk of hospitalization.

## AUTHOR CONTRIBUTIONS

Conceptualization: NK, NS, DRT and CFS; investigation: NJ and PB; methodology: NK, NS, DRT and CFS; validation: NK, NS, DRT and CFS; writing original draft: NK and NS; writing review and editing: DRT and CFS; visualization: NK, NS, DRT and CFS.

## FUNDING INFORMATION

This research received no specific grant from any funding agency in the public, commercial, or not‐for‐profit sectors.

## CONFLICT OF INTEREST STATEMENT

All authors declare that there are no conflicts of interest.

## Supporting information


Data S1:

## Data Availability

The data that support the findings of this study are available from the corresponding author upon reasonable request.
