# Supplementary material for: Clinical and demographic moderators of self‐care and hospitalizations in pre‐coronary artery bypass grafting patients: A cross‐sectional study
Source: J Clin Nurs. 2024 Aug 6;34(6):2171–80. doi: 10.1111/jocn.17381 (PMC12125537; doi:10.1111/jocn.17381)
Supplement: Supplementary file 1 — Data S1: [file JOCN-34-2171-s001.doc]

STROBE Statement—Checklist of items that should be included in reports of ***cross-sectional studies***

|  | Item No | Recommendation |
| --- | --- | --- |
| **Title and abstract** | 1 | (*a*) Indicate the study’s design with a commonly used term in the title or the abstract  Cross-sectional study as stated in the Title and Abstract on page 1,2 and Methods on page 6. |
|  |  | (*b*) Provide in the abstract an informative and balanced summary of what was done and what was found  Provided in Abstract on page 2. |
| Introduction | | |
| Background/rationale | 2 | Explain the scientific background and rationale for the investigation being reported  Included in the Introduction on pages 4-6. |
| Objectives | 3 | State specific objectives, including any prespecified hypotheses  Included in the Introduction on page 6. |
| Methods | | |
| Study design | 4 | Present key elements of study design early in the paper  Provided in the Study design on page 6. |
| Setting | 5 | Describe the setting, locations, and relevant dates, including periods of recruitment, exposure, follow-up, and data collection  Included in the Methods on pages 6-9. |
| Participants | 6 | (*a*) Give the eligibility criteria, and the sources and methods of selection of participants  Provided in the 2.2 Sample on page 7. |
| Variables | 7 | Clearly define all outcomes, exposures, predictors, potential confounders, and effect modifiers. Give diagnostic criteria, if applicable  Included in the Methods on pages 6-9. |
| Data sources/ measurement | 8* | For each variable of interest, give sources of data and details of methods of assessment (measurement). Describe comparability of assessment methods if there is more than one group  Included in the Methods on pages 6-9. |
| Bias | 9 | Describe any efforts to address potential sources of bias  Addressed in the Discussion on page 16. |
| Study size | 10 | Explain how the study size was arrived at  Provided in 2.5 Sample size calculation on page 9. |
| Quantitative variables | 11 | Explain how quantitative variables were handled in the analyses. If applicable, describe which groupings were chosen and why  Included in the Methods on pages 6-10. |
| Statistical methods | 12 | (*a*) Describe all statistical methods, including those used to control for confounding  Provided in 2.6 Statistical analysis on page 9-10. |
|  |  | (*b*) Describe any methods used to examine subgroups and interactions  Provided in 2.6 Statistical analysis on page 9-10. |
|  |  | (*c*) Explain how missing data were addressed  Included in the Methods on pages 6-9. |
|  |  | (*d*) If applicable, describe analytical methods taking account of sampling strategy  Not applicable. |
|  |  | (*e*) Describe any sensitivity analyses  Not applicable. |
| Results | | |
| Participants | 13* | (a) Report numbers of individuals at each stage of study—eg numbers potentially eligible, examined for eligibility, confirmed eligible, included in the study, completing follow-up, and analysed  Included in the Results on page 11. |
|  |  | (b) Give reasons for non-participation at each stage  Not applicable. |
|  |  | (c) Consider use of a flow diagram  Not applicable. |
| Descriptive data | 14* | (a) Give characteristics of study participants (eg demographic, clinical, social) and information on exposures and potential confounders  Included in the Results on page 11 and Table 1 on page 17. |
|  |  | (b) Indicate number of participants with missing data for each variable of interest  Included in the Results on page 11 and Table 1 on page 17. |
| Outcome data | 15* | Report numbers of outcome events or summary measures  Included in the Results on pages 11-13 |
| Main results | 16 | (*a*) Give unadjusted estimates and, if applicable, confounder-adjusted estimates and their precision (eg, 95% confidence interval). Make clear which confounders were adjusted for and why they were included  Included in the Results on pages 11-13 |
|  |  | (*b*) Report category boundaries when continuous variables were categorized  Not applicable. |
|  |  | (*c*) If relevant, consider translating estimates of relative risk into absolute risk for a meaningful time period  Not applicable. |
| Other analyses | 17 | Report other analyses done—eg analyses of subgroups and interactions, and sensitivity analyses  Not applicable |
| Discussion | | |
| Key results | 18 | Summarise key results with reference to study objectives  Included in the Discussion on page 14. |
| Limitations | 19 | Discuss limitations of the study, taking into account sources of potential bias or imprecision. Discuss both direction and magnitude of any potential bias  Included in the Discussion on page 16. |
| Interpretation | 20 | Give a cautious overall interpretation of results considering objectives, limitations, multiplicity of analyses, results from similar studies, and other relevant evidence  Included in the Discussion on page 14-16. |
| Generalisability | 21 | Discuss the generalisability (external validity) of the study results  Included in the Discussion on page 14-16. |
| Other information | | |
| Funding | 22 | Give the source of funding and the role of the funders for the present study and, if applicable, for the original study on which the present article is based  Provided in the Title page. |

*Give information separately for exposed and unexposed groups.

**Note:** An Explanation and Elaboration article discusses each checklist item and gives methodological background and published examples of transparent reporting. The STROBE checklist is best used in conjunction with this article (freely available on the Web sites of PLoS Medicine at http://www.plosmedicine.org/, Annals of Internal Medicine at http://www.annals.org/, and Epidemiology at http://www.epidem.com/). Information on the STROBE Initiative is available at www.strobe-statement.org.
